# Evaluation of Antimicrobial Activities against Various *E. coli* Strains of a Novel Hybrid Peptide—LENART01

**DOI:** 10.3390/molecules28134955

**Published:** 2023-06-23

**Authors:** Pawel Serafin, Paweł Kowalczyk, Adriano Mollica, Azzurra Stefanucci, Anna K. Laskowska, Magdalena Zawadzka, Karol Kramkowski, Patrycja Kleczkowska

**Affiliations:** 1 Military Institute of Hygiene and Epidemiology, 01-163 Warsaw, Poland; pawelserafin1@wp.pl (P.S.); magdalena.zawadzka@wihe.pl (M.Z.); 2 Department of Animal Nutrition, The Kielanowski Institute of Animal Physiology and Nutrition, Polish Academy of Sciences, 05-110 Jabłonna, Poland; p.kowalczyk@ifzz.pl; 3 Department of Pharmacy, University “G. d’Annunzio” of Chieti-Pescara, Via dei Vestini 31, 66100 Chieti, Italy; a.mollica@unich.it (A.M.); a.stefanucci@unich.it (A.S.); 4 Centre for Preclinical Research and Technology (CePT), Department of Pharmaceutical Microbiology, Faculty of Pharmacy, Medical University of Warsaw, 02-097 Warsaw, Poland; ak.laskowska@yahoo.com; 5 Department of Epidemiology and Public Health Lodz, Medical University of Lodz, 90-419 Lodz, Poland; 6 Department of Physical Chemistry, Medical University of Bialystok, 15-089 Białystok, Poland; kkramk@wp.pl; 7 Maria Sklodowska-Curie Medical Academy in Warsaw, 03-411 Warsaw, Poland

**Keywords:** antimicrobial activity, *E. coli* cells, MIC, MBC, hybrid peptide, opioids, antimicrobial activity

## Abstract

Finding the ideal antimicrobial drug with improved efficacy and a safety profile that eliminates antibiotic resistance caused by pathogens remains a difficult task. Indeed, there is an urgent need for innovation in the design and development of a microbial inhibitor. Given that many promising antimicrobial peptides with excellent broad-spectrum antibacterial properties are secreted by some frog species (e.g., bombesins, opioids, temporins, etc.), our goal was to identify the antimicrobial properties of amphibian-derived dermorphin and ranatensin peptides, which were combined to produce a hybrid compound. This new chimera (named LENART01) was tested for its antimicrobial activity against *E. coli* strains K12 and R1–R4, which are characterized by differences in lipopolysaccharide (LPS) core oligosaccharide structure. The results showed that LENART01 had superior activity against the R2 and R4 strains compared with the effects of the clinically available antibiotics ciprofloxacin or bleomycin (MIC values). Importantly, the inhibitory effect was not concentration dependent; however, LENART01 showed a time- and dose-dependent hemolytic effect in hemolytic assays.

## 1. Introduction

The resistance of bacterial cells to environmental conditions, such as antibiotic exposure, is now becoming an increasing problem. Many studies have shown that resistant bacteria carry the risk of serious health problems, including dysfunction of major human organs and tissues and increased mortality [1,2,3,4]. Moreover, they generate the need to develop drug after drug. This resistance phenomenon is primarily a consequence of the presence of a common enterobacterial antigen (ECA), which appears to be closely related to lipopolysaccharide (LPS). In this context, the most abundant bacteria and strains of *Escherichia coli* have been found to produce specific anti-ECA antibodies [5]. This is true for *E. coli* strains K12 and R1–R4, which are characterized by differences in the structure of the LPS core oligosaccharide. Indeed, the usual LPS of an *E. coli* strain, known as a smooth molecule, usually consists of a tripartite structure containing both hydrophilic lipid A, a phosphorylated core oligosaccharide divided into an outer and inner core, and a polysaccharide, which forms the *O* antigen detected during serotyping. In contrast, the LPS of strain K12, as well as strains R1–R4, a so-called rough form, does not contain the *O* antigen due to mutations in the *O* antigen operon [6,7]. Consequently, the LPS structure of these *E. coli* strains plays a crucial role in membrane permeability [8] and thus has a major impact on the antimicrobial activity exerted by various commonly used drugs.

Since antibiotics are the only possible treatment, and their efficacy against many bacterial infections is declining, much attention has been paid to the discovery of a new generation of drugs. Among them, hybrid compounds seem to be a desirable candidate to solve the above problems. This is because the hybrid approach aims to improve treatment efficacy and reduce the development of drug resistance. Such structures consist of two different drugs combined in a single molecule, and this combination makes them available to overcome clinically significant side effects, such as toxicity [9,10,11].

It has been reported that amphibian skin contains a rich arsenal of compounds with potent antimicrobial activity [12,13]. These include opioids and bombesin-like peptides [14,15,16]. Unfortunately, information on the antimicrobial activity of bombesin is limited. However, bombesin has shown efficacy in reducing bacterial translocation [17]. In turn, some opioids have shown antimicrobial properties [18]. In addition, morphine, a natural exogenous alkaloid to the μ-opioid receptor, stimulates the immune system by binding to myeloid differentiation protein 2 (MD2), a molecule associated with toll-like receptor 4 (TLR4), the receptor for bacterial lipopolysaccharide (LPS). Wright et al. also suggested that opioids may be recognized by bacteria as signaling molecules [14].

**Figure 1:**
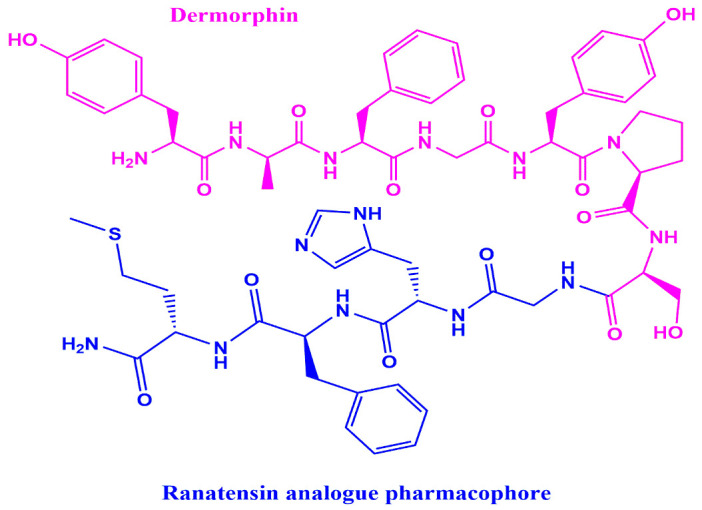
Chemical structure of LENART01 (YdAFGYPSGHFM), an opioid–ranatensin hybrid peptide.

Given this, the purpose of this study was to determine the antimicrobial activity of a novel hybrid peptide LENART01 (Figure 1), which is composed of two peptides derived from the skin of a frog against *E. coli* strains. LENART01 is characterized in that dermorphin, a heptapeptide isolated from the skin of the frog *Phyllomedusa sauvagei*, is covalently linked to ranatensin (RAN), an undecapeptide first isolated from the skin of the frog *Rana pipiens* in 1970 [19,20,21]. While dermorphin is a known potent μ-opioid receptor agonist with a specific amino acid sequence with D-Ala^2^ located at the N-terminus [22], RAN is homologous to bombesin (BN) [23]. Both peptides have diverse effects, including effects on blood pressure, as well as analgesic effects [22,23]. However, there is no information in the literature on their antimicrobial activity, either alone or as a hybrid.

## 2. Results

### 2.1. LENART01 Displayed Antimicrobial Activity against K12 and R2–R4 Model Strains of E. coli That Differ in LPS Structure

**Figure 2:**
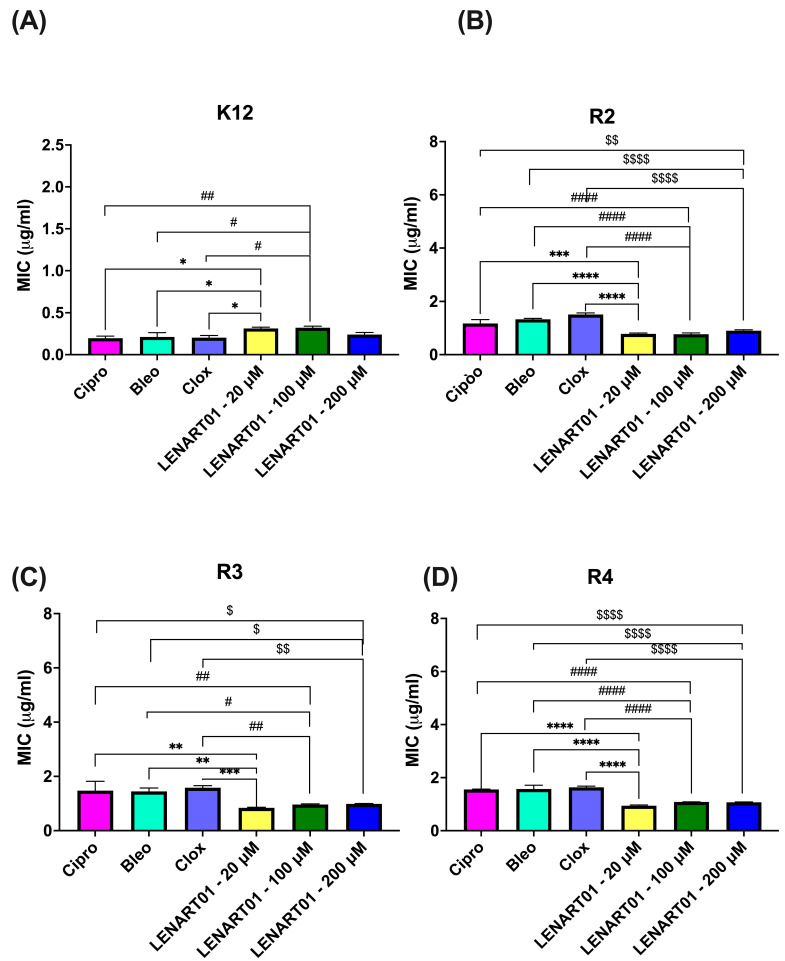
Minimum inhibitory concentration (MIC) of LENART01 and selected clinically available antibacterial agents in analyzed model bacterial strains: (**A**) K12; (**B**) R2; (**C**) R3, and (**D**) R4. LENART01 was administered at three different doses of 20, 100, and 200 μM. The results were compared with ciprofloxacin (Cipro; 10 mM/mL), bleomycin (Bleo; 10 mM/mL), and cloxacillin (Clox; 10 mM/mL). One-way ANOVA with Tukey’s post hoc test revealed significant differences between LENART01 and antibiotics (* *p* < 0.05, ** *p* < 0.01, *** *p* < 0.001, **** *p* < 0.0001; # *p* < 0.05, ## *p* < 0.01, #### *p* < 0.0001; $ *p* < 0.05, $$ *p* < 0.01, $$$$ *p* < 0.0001), with *—for 20 μM LENART01, #—for 100 μM, and $—for 200 μM LENART01, respectively. No significant differences were noted between LENART01 at various concentrations (*p* > 0.05).

**Figure 3:**
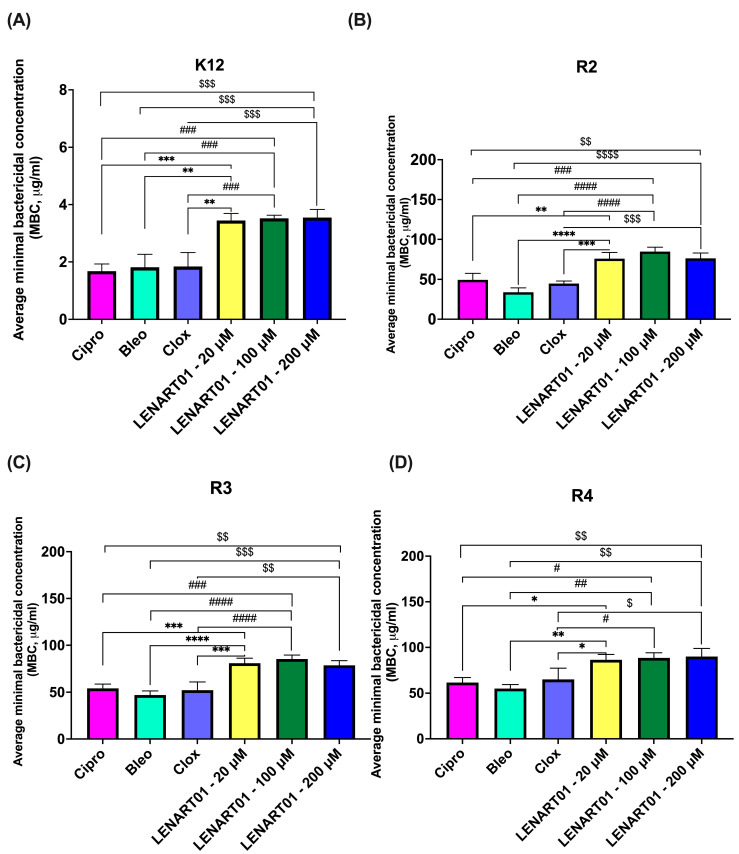
Minimum bactericidal concentration (MBC) of LENART01 and selected clinically available antibacterial agents in analyzed model bacterial strains: (**A**) K12; (**B**) R2; (**C**) R3, and (**D**) R4. LENART01 was administered at three different doses of 20, 100, and 200 μM. The results were compared with ciprofloxacin (Cipro; 10 mM/mL), bleomycin (Bleo; 10 mM/mL), and cloxacillin (Clox; 10 mM/mL). One-way ANOVA with Tukey’s post hoc test revealed significant differences between LENART01 and antibiotics (* *p* < 0.05, ** *p* < 0.01, *** *p* < 0.001, **** *p* < 0.0001; # *p* < 0.05, ## *p* < 0.01, ### *p* < 0.001, #### *p* < 0.0001; $ *p* < 0.05, $$ *p* < 0.01, $$$ *p* < 0.001, $$$$ *p* < 0.0001), with *—for 20 μM LENART01, #—for 100 μM, and $—for 200 μM LENART01. No statistics were observed between LENART01’s various concentrations (*p* > 0.05).

The results of the antimicrobial activity induced by LENART01 are shown in Figure 2 and Figure 3. The chimera demonstrated potent antibacterial activity against R2, R3, and R4 *E. coli* strains with MICs of 0.782–0.899, 0.839–0.988, and 0.942–1.070 μg/mL, respectively (Figure 2B–D). Interestingly, compared to microbial inhibitors (i.e., ciprofloxacin, bleomycin, and cloxacillin) administered at higher concentrations (10 mM/mL), 200 μM LENART01 proved almost as active (with no statistical differences) against strain K12 (Figure 2A), while low concentrations of the compound (20 or 100 μM) resulted in significantly weaker antimicrobial activity when the K12 strain was used. LENART01, on the other hand, showed higher activity, particularly on R2 and R4 strains (Figure 2B,D), compared to the effects of Cipro or Bleo. It is worth noting that, in the case of the R2 and R3 strains, the control compounds seemed to show no inhibitory activity against *Escherichia coli* at the concentration used (Figure 2B,C).

According to MBC, all *E. coli* strains tested were susceptible to the LENART01 chimera at every dose used in the study. However, the bactericidal effect was significantly lower compared to the results for Cipro, Bleo, or Clox. In fact, the MBC values were higher for LENART01, ranging from 3.443 to 3.547 μg/mL for the K12 strain, 75.833 to 84.666 μg/mL for the R2 strain, 78.566 to 85.333 μg/mL for the R3 strain, and 86.400 to 90.000 μg/mL for the R4 strain (Figure 3A–D; significant differences are shown in the figure legend). Interestingly, neither the highest nor the lowest concentration of LENART01 resulted in a significant change in its bactericidal activity (*p* > 0.05). Therefore, the effect was not concentration dependent (Figure 3A–D).

The MBC/MIC ratio is known to be useful in determining the nature of antimicrobial activity induced by a compound. In this regard, it is known that an MBC/MIC ratio below 4 indicates bactericidal activity of the compounds, while, with an MBC/MIC ratio > 4, a drug is considered bacteriostatic [24,25]. In our studies, LENART01 showed bacteriostatic activity regardless of the concentration used (Table 1). These values also indicated the occurrence of pathogen tolerance to the drug under the given conditions.

### 2.2. Contribution of LENART01 Pharmacophores to Antimicrobial Effect Exerted on Model Strains of E. coli

Since the test compound consisted of two different pharmacophores that contained an opioid- and bombesin-related structural unit, and *E. coli* exhibits an expression of opioid receptors, the purpose of the experiment was to determine the contribution of the opioid receptor system to the antimicrobial activity exerted by LENART01.

**Figure 4:**
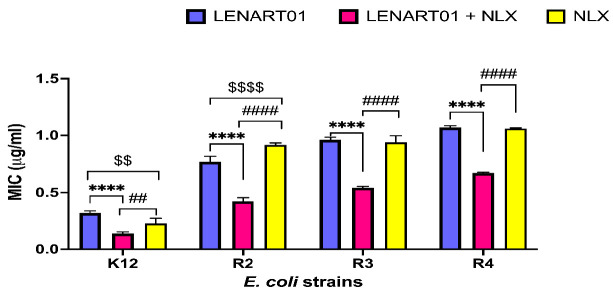
MIC results for LENART01 (100 μM) administered with naloxone (NLX) in model E. coli strains. Two-way ANOVA followed by Bonferroni’s post hoc test showed significant differences (**** *p* < 0.0001) between LENART01 and LENART01 inhibited with an opioid antagonist—NLX. In addition, significant differences were found for LENART01 + NLX vs. NLX (## *p* < 0.01 for strain K12 and #### *p* < 0.0001 for strains R2–R4) and LENART01 vs. NLX ($$ *p* < 0.01 for strain K12 and $$$$ *p* < 0.0001 for strain R2).

As shown below, inhibition of LENART01 (100 μM/mL) with NLX resulted in a significant reduction in MIC values by almost half for all model strains, K12, R2, R3, and R4, analyzed. For example, while the 0.321 μg/mL ± 0.011 value of LENART01 proved effective in inhibiting bacterial growth, simultaneous application of NLX reduced the MIC to 0.138 ± 0.009 (for the K12 strain; Figure 4). Similar results were observed for other strains: (i) from 0.768 μg/mL ± 0.028 of LENART01 to 0.424 μg/mL ± 0.018 for LENART01 + NLX (for strain R2); (ii) from 0.961 μg/mL ± 0.014 of LENART01 to 0.542 μg/mL ± 0.007 for LENART01 + NLX (for strain R3); and (iii) from 1.085 μg/mL ± 0.003 of LENART01 to 0.671 μg/mL ± 0.005 for LENART01 + NLX (for strain R4).

### 2.3. Modification of Plasmid DNA Isolated from E. coli R2–R4 Strains Recognized with Fpg Protein

**Figure 5:**
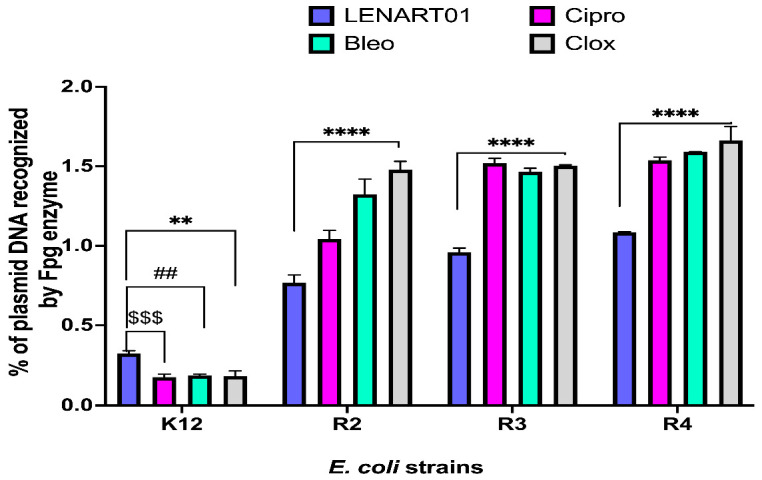
Percentage of plasmid DNA recognized by Fpg enzyme with model bacterial, K12, and R2–R4 strains. Two-way ANOVA followed by Tukey’s post hoc test revealed significant differences between LENART01 and antibiotics (Cipro, Bleo, and Clox) in every *E. coli* strain (** *p* < 0.01, **** *p* < 0.0001; ## *p* < 0.01; $$$ *p* < 0.001), where * is for LENART01 vs. Clox, #—LENART01 vs. Bleo, and $—LENART01 vs. Cipro; no significant differences were observed between microbial inhibitors used in the case of K12, R2, and R3, while, in R4—** *p* < 0.01 for Cipro vs. Clox (Data not shown in the figure).

As can be observed, bacterial plasmid DNA digested with Fpg enzyme modified with clinically available antibiotics exhibited significant differences compared to the LENART01 (Figure 5). Indeed, Cipro, Bleo, or Clox were found to cause more severe damage to *E. coli* strains R2, R3, and R4 than the hybrid peptide, as the percentage of plasmid DNA damage detected by Fpg was as follows: 1.042–1.539% (for Cipro), 1.324–1.591% (for Bleo), and 1.480–1.663% (for Clox) vs. 0.768–1.085% (LENART01) (Figure 5).

### 2.4. Hemolytic Effect

**Figure 6:**
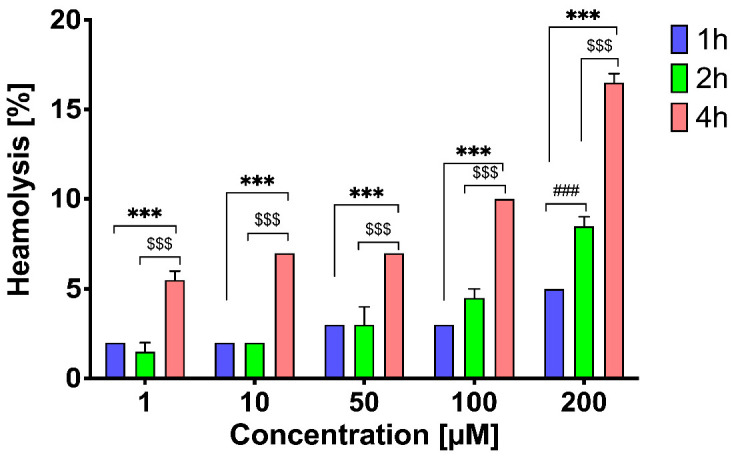
Time- and dose-dependent hemolytic activity of LENART01. Two-way ANOVA with Bonferroni’s post hoc test revealed significant differences between each concentration at each time point (*** *p* < 0.001, ### *p* < 0.001, and $$$ *p* < 0.001), where * is for 1 h vs. 4 h, $ is for 2 h vs. 4 h, and # is for 1 h vs. 2 h.

The hemolytic activity of the peptide was tested to determine its potential toxicity. The hemolytic effect of LENART01 on RBC was measured after 1, 2, and 4 h of incubation. Hemolysis induced by the peptide was time and concentration dependent (*p* < 0.001). The lowest level of hemolysis was observed after 1 h of exposure to the peptide, with a maximum of 4.8% at a concentration of 200 µM. However, as incubation time increased, an increase in induced hemolysis was observed at all concentrations tested (Figure 6). After 2 and 4 h of incubation, the maximum level of hemolysis was 8.3% and 16.4% at the 200 µM concentration, respectively. The hemolysis values measured at each time point were statistically significant. The greatest difference in hemolysis was observed between 1 h and 4 h of incubation in all case studies (*p* < 0.001).

## 3. Discussion

The search for an effective drug that combines the desired potent antibiotic activity with an acceptable safety profile and a low risk of developing antimicrobial resistance (AMR) is still ongoing. In this context, several types of amphibian-secreted peptides have recently returned to favor. However, little or nothing has been reported on animal opioid peptides and bombesin.

The literature data on antimicrobial effects mediated by opioids are inconclusive. In fact, Rosenberg and Renkonen showed that morphine does not affect microbial growth when administered in the concentration range of 0.2 to 2 mg/mL [27]; however, some work contradicts this. This includes the work of Unlu et al. [28], in which tramadol and fentanyl showed dose-dependent inhibitory effects on various bacteria, including *E. coli*. Others compared bupivacaine and pethidine, demonstrating their antimicrobial activity [29]. Consequently, Mami and colleagues [30] demonstrated the antibacterial effect of opium (*Papaver somniferum*). Unfortunately, to date, no studies have been conducted on the antimicrobial inhibitory activity of opioid peptides derived from frog skin, such as dermorphin.

Similar positive results have been reported for bombesin [15,31], but, again, there is a lack of information on bombesin-related peptides (such as ranatensin, phyllolitorin, or litorin) secreted from amphibian skin.

Therefore, the present study aimed to investigate the antimicrobial activity of the novel opioid-based hybrid peptide LENART01 against Gram-negative bacteria *E. coli,* with a focus on strains that differ in LPS structure, which is crucial for membrane permeability [8].

The hybridization strategy is based on the design and development of a compound that combines two different biologically active molecules that act on different targets so that the effect produced by each molecule is also combined (e.g., an additive effect, synergistic effect, etc.) [32,33]. Hybrid structures, known as multi-target ligands or multifunctional compounds, are also known to reduce toxicity [34] and thus have lower adverse side effects and better pharmacokinetics [35,36,37]. In this case, the combination of dermorphin and ranatensin derivatives should yield a potent compound with the desired safety profile. The study conducted by our group showed that LENART01 is a biologically active compound with potent antimicrobial activity against *E. coli* strains at concentrations much lower (µM range) than clinically available antibiotics (i.e., 10 mM of Cipro, Bleo, and Clox). Although no statistical differences in MIC or MBC values were observed between different concentrations of LENART01 (*p* > 0.05) (Figure 2 and Figure 3, respectively), the MICs of the chimera were significantly lower than those of the aforementioned antibiotics against all bacterial strains tested, except for strain K12 (Figure 2). In fact, MICs ranged from 0.782 to 1.070 μg/mL, better than Cipro (from 1.170 to 1.553), Bleo (from 1.327 to 1.573), and Clox (from 1.510 to 1.633) (Figure 2B–D). This effect may have been due to the specific design of the chimera, as LENART01 contains a *D*-alanine residue at the second position in the N-terminal dermorphin [38,39]. In line with this, Cava et al. [40] found that *D*-amino acids play a key role in cell wall remodeling and biofilm degradation in the bacterial kingdom. They also found that they serve as nutrients that promote bacterial growth [41]. Furthermore, for example, *D*-serine, the most abundant amino acid in human urine, has been found to alter the gene expression of uropathogenic *E. coli* [42]. Nevertheless, despite the undeniable role of amino acids in the spread of bacterial infections and bacterial drug resistance, *D*-amino acids in particular have been shown to possess a broad spectrum of antimicrobial properties [43,44,45]. Most antibiotics act on bacteria by inhibiting cell wall synthesis, with peptidoglycan (PG) being the primary target [46]. Although the exact mechanism of the bactericidal action exhibited by LENART01 remains unknown, we hypothesize that it may also be related to PG disruption. In this regard, since bacteria are known to produce and effectively use various enzymes to modify or destroy antibiotics [47], they may damage the structure of LENART01 itself to generate various products of its disruption, including single amino acids. Bacteria can produce additional *D*-amino acids through the racemization of both proteinogenic and non-proteinogenic *L*-amino acids [48]. Since LENART01 contains a serine residue, it is hypothesized that Ser can be converted to *D*-Ser. Importantly, *D*-Ser may be responsible for the attenuating transpeptidation by replacing the *D*-Ala–*D*-Ala bonding with a *D*-Ala–*D*-Ser bond, as noted by Wang et al. [49]. This re-substitution of *D*-Ala in the peptidoglycan layer may weaken its thickness and thus make the cell vulnerable to the peptide or its derivatives.

Another possible explanation for the observed effect is related to bacterial transcription. In this context, bacterial LPS is known to be regulated by several positive transcriptional factors, including RpoE and RfaH [50,51]. RfaH is a two-domain protein with a C-terminal domain in the α-helical state which can switch reversibly between the α-helix and the β-barrel [52]. Through direct interactions with RNA polymerase (RNAP) and ribosome, RfaH activates cell wall and capsule expression by reducing transcription termination and activating translation [53]. More recently, *D*-amino acids have been confirmed to act as helix breakers [54]. Therefore, we can hypothesize that *D*-Ala LENART01, depending on the breaking position in RfaH, may interfere with RfaH function, thereby inhibiting its recruitment to RNAP and affecting the cell.

Of course, other potential mechanisms are also possible. For instance, LENART01 may behave at least similarly to the antimicrobial peptides (AMPs; e.g., temporins, bombinins, brevinins, etc.) widely secreted by many amphibian species. However, further studies should be conducted to compare the hybrid with AMPs. These should include the determination/calculation of charge, length, hydrophobicity, and the ratio between hydrophobic and charged amino acids; these parameters are crucial for the design of potent antimicrobial compounds and have been suggested to serve as a determinant of the spectrum of peptide activity [55,56,57,58].

As mentioned earlier, opioid ligands have been reported to interact with TLRs, which are known for their dominant role in recognizing pathogen infection. Moreover, TLRs, especially TLR4, are receptors for bacterial LPS. Thus, it triggers an innate immune response as a result of LPS stimulation [59]. In this context, it has been suggested that the antimicrobial activity induced by LENART01 depends on its structural components, as its co-administration with NLX resulted in a significant decrease in MIC values (**** *p* < 0.0001), which are necessary to achieve the desired effect (Figure 4). Indeed, NLX was found to target the LPS binding pocket of MD-2 and block the innate immune TLR4 signaling [60]. However, similar results have been reported for opioid agonists [61]. Subsequently, Stevens and colleagues [62] reported that both opioid agonists and antagonists inhibit LPS signaling in a non-competitive manner through opioid site(s) other than GPCR in the TLR4 signaling pathway. This was also confirmed in our studies, which showed that both the chimera and NLX had almost the same efficacy when administered separately. Therefore, neither LENART01 nor NLX proved to be a more effective ligand for TLR4. Since the concomitant use of both drugs exerts a lower MIC, there is likely a synergistic relationship between them. Nevertheless, to uncover the specific mechanism of this phenomenon, the involvement of ranatensin in the overall activity should first be established.

Fpg, also known as 8-oxoguanine DNA glycosylase, releases damaged purines from DNA. However, because it has low specificity, it can recognize and remove other damaged bases, including pyrimidines [63,64,65]. Nevertheless, we observed a low percentage of plasmid DNA damage detected by Fpg after exposure to LENART01 (Figure 5). This was contrary to the effect mediated by the antibiotics used, as Cipro, Bleo, and Clox increased the number of lesions in every *E. coli* strain except K12 (Figure 5). This may indicate that DNA modified with the aforementioned inhibitors and digested with Fpg protein yield substrates whose structures are similar to those of purines and pyrimidines. On the contrary, the results obtained for the chimera were probably due to (i) less DNA damage induced in the plasmid DNA treated with the peptide or (ii) the absence or a small number of specific Fpg-sensitive sites on the plasmid molecule. Therefore, it is assumed that the LENART01 chimera causes little oxidative damage. Therefore, it is suggested that the proposed mechanism of its antimicrobial action is non-oxidative.

An ideal drug should have high therapeutic activity and high safety and therefore have relatively few or no side effects. Preliminary results of toxicity studies, including the hemolytic activity of the LENART01 chimera, are presented.

It can be observed that the hemolytic effect induced by the peptide increased with increasing incubation time (Figure 6). Only concentrations of 1 and 10 μM (incubation up to 2 h) can be considered promising, as substances/compounds showing hemolysis of 2.5% or less are considered safe for intravenous administration [66]. However, as the concentration and incubation time increased, LENART01 showed its toxic potential. This undesirable activity was particularly pronounced at a concentration of 200 µM. Importantly, LENART01 behaved similarly to AMPs in this case. In fact, AMPs were found to be more hemolytic with increasing hydrophobicity, a property evident in the antimicrobial activity of this type of compounds, which correlates with both charge and hydrophobicity [67]. The more polar the peptide, the more often it exhibits no or negligible hemolytic activity. Nevertheless, since LENART01 is unstable, as our preliminary studies on its stability showed that the peptide is short acting, with a short half-life (project in progress), we can assume that further exposure of blood cells to its action could result in a constant hemolysis value. To complete the study, LENART0-induced hemolytic activity should also be investigated in vivo, followed by a toxicity dose range study, as the correlation between the two is not obvious [68].

Unfortunately, the overall results obtained in our studies cannot be compared with those obtained by other groups on the antimicrobial activity of hybrid peptides, because so far there is no hybrid structure comparable to the type of pharmacophores used. In fact, most chimeras containing dermorphin as an N-terminal element have been tested for their analgesic activity, as this opioid is a more potent and selective compound with long-lasting analgesia compared to morphine [19,69]. Similarly, it seems difficult to compare its activity with that of bombesin. Intriguingly, ranatensin, used here as a modified C-terminal pharmacophore of the chimera, is characterized by a different molecular target than bombesin (dopaminergic receptors vs. bombesin receptors, respectively; [70]).

## 4. Conclusions

The results obtained confirmed that LENART01, a novel opioid–bombesin-based hybrid peptide, is highly effective against various strains of *E. coli* in vitro. In addition, this compound has a low or moderate hemolytic activity (especially at a concentration of 1 and 10 μM; incubation up to 2 h). LENART01 exerts little damage to plasmid DNA. Therefore, it does not induce an SOS response or increase bacterial mutation rate, which, in consequence, may prevent the resistance development of microbial pathogens. Collectively, these features make LENART01 an interesting and promising candidate for the development of a new class of peptide antibiotics.

## 5. Materials and Methods

### 5.1. Reagents and Microorganisms

For peptide synthesis and purification, all reagents and solvents were from Merck (Milano, Italy).

For antimicrobial activity determination, *Escherichia coli* K12 and R1–R4 strains were received from Prof. Jolanta Łukasiewicz at the Ludwik Hirszfeld Institute of Immunology and Experimental Therapy (Polish Academy of Sciences, Warsaw, Poland). The reference bacterial strains of *E. coli* (K12 ATCC 25404, R2 ATCC 39544, R3 ATCC 11775, R4 ATCC 39543) were provided by LGC Standards (Lancashire, UK), and were used according to the recommendation of ISO 11133: 2014 [71]. *N*,*N*-dimethylformamide (DMF) was purchased from Sigma-Aldrich (Poznań, Poland), while the Lanes 1kb ladder and Quick Extend DNA ladder were from New England Biolabs (Ipswich, MA, USA).

### 5.2. Synthesis and Purification of LENART01, an Opioid–Ranatensin Hybrid Peptide

The desired peptide, namely LENART01, was prepared as a *C*-terminal amide following a well-established solid-phase peptide synthesis protocol [11]. Fmoc-protected amino acids were purchased from Sigma-Aldrich (Milano, Italy). Boc-protected and *tert*-butyl-protected side chains were selected for His and Tyr/Ser respectively, together with a Rink amide resin with loading coefficient 1.2 mM/g.

The crude peptide was obtained by using the coupling cocktail TBTU/HOBt/DIPEA and the Fmoc removal solution of piperidine 20% in DMF. Once the sequence was completed, the peptide was cleaved from the solid support using a cocktail of TFA/H_2_O/TIPS, = 95:2.5:2.5, triturated by cold ether (five times), dried under vacuum, and purified via RP-HPLC following the procedure previously described by us and briefly reported here [72].

The crude peptide LENART01 was purified in RP-HPLC using a Waters XBridge Prep BEH130 C18 column, 5.0 μm, 250 mm × 10 mm, at a flow of 5 mL/min, and a Waters 600 binary pump (Milford, MA, USA), using as eluent a linear gradient of H_2_O/ACN 0.1% TFA from 5% ACN to 90% ACN in 35 min. The purity of LENART01 was checked by analytical RP-HPLC at 220 nm using a Kromasil 100-5C18 column, 5.0 μm, 250 mm × 4.6 mm, at a flow of 1 mL/min, using a gradient of H_2_O/ACN 0.1% TFA from 28% to 53% ACN in 20 min, and was found to be ≥ 97.7% (rt = 8.10 min). LRMS was performed on a LCQ Finnigan MAT mass spectrometer (San Jose, CA, USA) by ESI-spray source and ion trap analyzer, capillary temperature at 200 °C, the spray voltage at 3.00 kV. Nitrogen (N_2_) and helium as both the sheath gas and the auxiliary gas.

### 5.3. In Vitro DNA Damage by LENART01

Plasmids isolated from K12, R2, R3, and R4 *E. coli* bacterial strains were isolated by alkaline lysis, as described by Sambrook et al. [73]. The plasmid was reacted with 10 mM of analyzed control compounds (pH 5.5 for 16 h at 37 °C). After the reaction was finished, DNA isolated from analyzed strains was precipitated with ethanol 70% according to the standard procedure [74], washed, resuspended in sterile water, and stored at −20 °C until another analysis. LENART01-induced plasmid DNA damage was evaluated at concentrations of 20, 100, and 200 μM/mL in water. Next, the modified DNA obtained from the samples tested was digested with Fpg enzymes individually of glycosylase and AP endonuclease activity. The standard reaction mixture (final volume of 20 μL) for the Fpg protein consisted of 10 μg plasmid DNA, 0.09 μg Fpg/sample, 70mM Hepes–KOH (pH 7.8), 1 mM EDTA, 5 mM β-mercaptoethanol, 100 mM KCl, 100 μg/mL BSA, and 5% glycerol. Incubation was carried out at 37 °C for 30 min.

After cleavage of the plasmid DNA with DNA glycosylases and AP endonuclease, the enzymes were removed by chloroform extraction. DNA was further precipitated with 4 volumes of cold 96% ethanol with 0.1 volume of 3 M sodium acetate (pH 5.2, kept at −20 °C overnight or at −80 °C for 2 h) in order to sediment all small DNA fragments; subsequently, centrifugation at 12,000 rpm for 15 min was performed. The DNA pellet was resuspended in water, and DNA concentration was measured on a Varian Cary 3E spectrophotometer with ADL News software (version AU-VIC-RE-PS-006; Varian, Sunnyvale, CA, USA). To ensure complete removal of protein, the λ_260_/λ_280_ ratio was kept at 1.8 to 2.0. The DNA solution was stored at −20 °C until further use [74]. The integrity of the modified and digested plasmid was also verified by agarose gel electrophoresis as the ratio of the covalently closed circular to open circular form of the plasmid. The same bacterial DNA was modified by equal concentrations of antibiotics such as ciprofloxacin (Cipro), bleomycin (Bleo), and cloxacillin (Clox) at 10 μM/mL and by a selected concentration of LENART01, an opioid–ranatensin hybrid peptide.

### 5.4. Minimum Inhibitory Concentration (MIC) and Minimum Bactericidal Concentration (MBC)

In order to obtain the compound-exerted antimicrobial effect, LENART01 chimera was administered at three different doses (20, 100, and 200 μM) and used for isolated bacterial DNA from the analyzed model *E. coli* strains and in the strains themselves in MIC and MBC tests. LENART01-induced activity was compared with that of a negative control (untreated *E. coli* strains). Moreover, positive control was provided which included bacteria treated either with Cipro, Bleo, or Clox. In addition, we used naloxone (NLX; 2 mM) to determine the possible involvement of an opioid-receptor-related pharmacophore and changes in the antimicrobial activity of LENART01.

The MIC and MBC, defined as the lowest concentration of a bacteriostatic agent, were determined by a microtiter plate method using sterile 48- or 96-well plates. Briefly, 50 μL of the analyzed solutions (LENART01 chimera and appropriate *E. coli* K12 and R1–R4 strains) were added to the first row of the plate. Then, 25 μL of sterile Tryptone Soya Broth (TSB) medium was added to the other wells, and serial dilutions were performed. After that, 200 μL of inoculated TSB medium containing resazurin (0.02 mg/mL) as an indicator was added to all wells. The TSB medium was inoculated with 10^6^ colony-forming units (CFU)/mL (approximately 0.5 McFarland units) of the bacterial strains. The plates were incubated at 30 °C for 24 h. Color changes from blue to pink or yellowish with turbidity were considered positive, and the lowest concentration at which no visible color change occurred was MIC according to Koszelewski et al. [75]. Each experiment (both MIC and MBC) was repeated at least three times.

To estimate MBC, a dehydrogenase activity measurement was determined by measuring the visible color changes of triphenyl tetrazolium chloride (TTC) to triphenyl formazan (TF). A 4 mM amount of dense culture (approximately 10^9^ CFU/mL) incubated in TSB medium at 25 °C for 24 h was placed in identical test tubes. LENART01 (and reference drugs) was then added to the test tubes until the mixture reached a final concentration of 10–250 mg/ mL. Then, the cultures were incubated at 30 °C for 1 h. The test tubes were then sealed with parafilm and incubated for 1 h at 30 °C in the dark. The lowest concentration at which no visible red color (formazan) appeared was taken as the MBC.

### 5.5. Hemolysis

LENART01-induced hemolysis was measured according to the described method. Briefly, blood samples were taken from healthy volunteers into K2-EDTA-coated tubes to prevent coagulation. Samples were centrifuged for 10 min at 2500 rpm at 4 °C. Levels of hematocrit and plasma were noted on the tube. The plasma was gently aspirated, and PBS (pH 7.4, room temperature) was added up to the marked level of plasma. The solution was mixed gently and centrifuged for 10 min at 2500 rpm. The washing step was repeated three times. RBC suspension was diluted in PBS to obtain a 10% and 2% suspension of RBC. The 2% RBC suspension was incubated with LENART01 (1–200 μM) in a 1:1 ratio for 1, 2, and 4 h at 37 °C. At each time point, samples were centrifuged at 4500 rpm for 5 min, and 100 μL of supernatant from each sample was transferred to a 96-well plate. The absorbance was measured at a wavelength of 540 nm. A value of 100% hemolysis was determined by incubation of 10% in distilled water (ratio 1:9). For negative control (0% hemolysis), 2% RBC suspension was incubated with PBS (ratio 1:1). The experiment was performed in triplicate. The value of chimera-induced hemolysis was calculated:

Hemolysis [%] = (A − A0%)/(A100% − A0%) × 100%,

where A—absorbance of the sample, A100%—absorbance of positive control (100% hemolysis), and A0%—absorbance of negative control (0% hemolysis).

Hemolysis assay was conducted under the approval of the Bioethics Committee—Commission for the Supervision of Research on People and Animals at CSK MSWiA in Warsaw (no. 67/2017).

### 5.6. Statistical Analysis

Data obtained from the in vitro studies are presented as mean ± S.E.M and were analyzed using ANOVA and Bonferroni’s or Tukey’s post hoc tests. *p*-values < 0.05 were considered statistically significant. Data were analyzed using GraphPad Prism 9 for Macintosh (GraphPad Software Inc., San Diego, CA, USA).

## Figures and Tables

**Table 1:** Mean MBC/MIC ratio for LENART01 and selected antibiotics. Values were determined according to Borkowski et al. [26].

*E. coli* Strain	Compounds					
	Ciprofloxacin (10 mM/mL)	Bleomycin (10 mM/mL)	Cloxacillin (10 mM/mL)	LENART01		
				20 μM/mL	100 μM/mL	200 μM/mL
K12	97	98	98.8	100	100	148
R2	245	213	211	240	260	274
R3	305	295	303	335	365	381
R4	365	360	355	390	410	430

## Data Availability

On request from those interested.
